# Association of Preoperative Physical Activity with Short- and Long-Term Outcomes in Patients Undergoing Palliative Resection for Metastatic Colorectal Cancer: An Inverse Probability of Treatment Weighting Analysis

**DOI:** 10.3390/cancers14030489

**Published:** 2022-01-19

**Authors:** Ching-Chung Cheng, I-Li Lai, Shu-Huan Huang, Wen-Sy Tsai, Pao-Shiu Hsieh, Chien-Yuh Yeh, Sum-Fu Chiang, Hsin-Yuan Hung, Jeng-Fu You

**Affiliations:** 1 Division of Colon and Rectal Surgery, Department of Surgery, Chang Gung Memorial Hospital at Linkou, College of Medicine, Chang Gung University, Taoyuan 333, Taiwan; b9302047@adm.cgmh.org.tw (C.-C.C.); ryane92a5@cgmh.org.tw (I.-L.L.); mr0946@cgmh.org.tw (S.-H.H.); wensy@adm.cgmh.org.tw (W.-S.T.); hsiehps@adm.cgmh.org.tw (P.-S.H.); chnyuh@cgmh.org.tw (C.-Y.Y.); sumfu@cgmh.org.tw (S.-F.C.); 2 Division of Colon and Rectal Surgery, Department of Surgery, New Taipei Municipal TuCheng Hospital, New Taipei City 236, Taiwan; hsinyuan@adm.cgmh.org.tw

**Keywords:** colorectal cancer, long-term survival, metabolic equivalent of task, physical activity, inverse probability of treatment weighting, short-term morbidity

## Abstract

**Simple Summary:**

Physical activity is linked to the risk and the prognosis of colorectal cancer. However, the impact of preoperative physical activity on postoperative short-term and long-term outcomes is limited. The aim of our study was to elucidate the relationship of preoperative physical activity and postoperative outcomes in metastatic colorectal cancer patients who underwent palliative resection. After the inverse probability of the treatment weighting process, the metabolic equivalent of task < 12 group had a higher postoperative morbidity rate and mortality rate. No significant difference was found in overall survival. In patients undergoing palliative resection for metastatic colorectal cancer, preoperative leisure-time physical activity with the metabolic equivalent of task ≥ 12 was associated with reduced short-term postoperative morbidity and mortality; however, no difference was detected in long-term survival.

**Abstract:**

A lack of physical activity is a generally accepted risk factor for colorectal cancer. However, research on the effect of preoperative physical activity on postoperative and long-term outcomes is limited, especially in patients with stage IV colorectal cancer who underwent palliative surgery. Patients who underwent bowel resection for stage IV primary colorectal cancer between January 1995 and December 2016 were retrospectively enrolled. A total of 2185 patients were divided into two groups according to preoperative leisure-time weekly physical activity as assessed by metabolic equivalent of task (MET) values: MET < 12 (*n* = 1845) and MET ≥ 12 (*n* = 340). Inverse probability of treatment weighting (IPTW) was used to reduce imbalance and selection biases between the two groups. After the IPTW process, the MET < 12 group showed a higher postoperative morbidity rate (18.7% vs. 10.6%; *p* < 0.001) and mortality rate (2.4% vs. 0.6%; *p* < 0.001) than the MET ≥ 12 group. No significant difference was found in overall survival. Weekly preoperative leisure-time physical activity with MET ≥ 12 was associated with reduced short-term postoperative morbidity and mortality in patients undergoing palliative resection for metastatic colorectal cancer. However, no difference was detected in long-term survival.

## 1. Introduction

Colorectal cancer (CRC) is the third most common cancer and the second most common cause of cancer-associated death worldwide [1]. Risk factors for nonhereditary CRC (93–95% of CRC cases [2]) are wide-ranging and include positive family history [3], long-term inflammatory bowel disease, type 2 diabetes [4], previous CRC or adenoma, male sex, and lifestyle (low physical activity [5], smoking [6], alcohol consumption [7], obesity [8], low-fiber diet, and red and processed meat ingestion [9]).

The relationship between physical activity and CRC risk is well known. One pooled cohort study including 750,000 adults demonstrated that 7.5–15 metabolic equivalent task (MET) hours/week of physical activity could reduce CRC risk by 8–14% in men [10]. Low physical activity can lead to obesity (a risk factor for CRC), other comorbidities, and general deconditioning. General deconditioning can further weaken functional capacity and hinder patients from undergoing CRC treatments, such as surgery, adjuvant chemotherapy, and chemoradiation. Higher physical activity levels have been shown to reduce short-term postoperative complications in patients with CRC, but reports on long-term CRC outcomes are limited. One meta-analysis showed that preoperative cardiopulmonary exercise testing could predict postoperative complications and the length of hospital stay, but the maximum follow-up duration was 1 year, and long-term outcomes were not mentioned [11]. Our previous study showed that patients with nonmetastatic CRC with a MET higher than 12 h/week had more favorable short- and long-term outcomes after curative-intent resection [12]. However, the impact of physical activity on patients with metastatic CRC who underwent palliative surgery has not yet been reported.

Therefore, we conducted a qualitative analysis of the relationships between preoperative physical activity levels and short- and long-term outcomes of patients with metastatic CRC who underwent palliative surgery at a tertiary referral center during a 22-year period.

## 2. Materials and Methods

Data on clinicopathological features were retrieved from the Colorectal Section Tumor Registry of Chang Gung Memorial Hospital. The Institutional Review Board approved this study (IRB No.201601428B0).

### 2.1. Patient Selection

**Figure 1:**
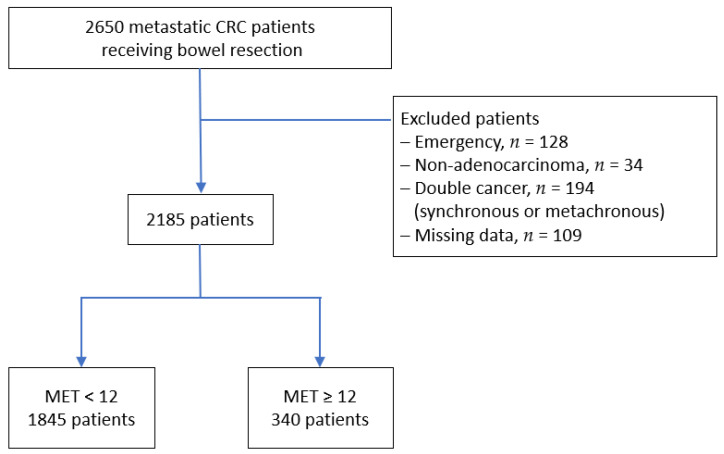
Flowchart of patient enrollment. CRC = colorectal cancer; MET = metabolic equivalent of task.

Between January 1995 and December 2016, 2650 patients at the referral center underwent bowel resection for stage IV primary CRC with or without metastasectomy. Of these 2650 patients, 12,834,194, and 109 were excluded because they had undergone emergency operations, had nonadenocarcinoma, had double cancer (either synchronous or metachronous cancer), or had unavailable data, respectively. The remaining 2185 patients were enrolled into this study (Figure 1). Data of preoperative variables (age, sex, body mass index [BMI], underlying disease, and carcinoembryonic antigen [CEA]), operative data (tumor location and type of surgery), postoperative data (type of complication and severity according to the Clavien–Dindo classification, postoperative hospital stay, pathological reports, and final clinical staging) and status of long-term follow-up were collected.

### 2.2. MET Values

MET value is the objective measure that estimates the energy expended by the body during physical activity compared to a reference and it can apply to people with varying body weights [13,14,15]. One MET is set by convention at 3.5 mL of oxygen per kilogram per minute and is approximately equivalent to the energy expended at rest or sitting idly. One of four nurse practitioners conducted a face-to-face interview with the patients following a prepared questionnaire to collect data in daily practice during admission. Weekly MET values of leisure-time activities were calculated according to the sum of exercises at least moderate intensity (MET ≥ 3) in a week. According to weekly MET values, 2185 patients were divided into two groups as follows: MET < 12 (*n* = 1845) and MET ≥ 12 (*n* = 340; Figure 1).

### 2.3. Inverse Probability of Treatment Weighting

An inverse probability of treatment weighting (IPTW) method was used to diminish the imbalance in potential confounding variables between the MET < 12 and MET ≥ 12 groups, with the possibility of bias arising owing to the difference in sample sizes and outcomes. To generate the underlying propensity score, baseline covariates included age, sex, hypertension, diabetes mellitus, tumor location (right colon, left colon, and rectum), T staging (T4 and non-T4), N staging (node-negative and node-positive), metastasis pattern (single and multiple organ), and chemotherapy (neoadjuvant and adjuvant) were used.

### 2.4. Outcomes and Covariables

Short-term postoperative complications and long-term survival were measured as patients’ outcomes. Morbidity occurring within 30 days after operation were regarded as postoperative complications. These complications were divided to wound-related complications, pulmonary-related complications, cardiovascular-related complications, urinary-related complications, GI-related complication, abdominal-related complications, anastomosis, and other rare complications. Death occurring within 30 days after surgery was regarded as postoperative mortality. Complications were rated in accordance with the Clavien–Dindo classification [16]. Long-term outcomes were assessed by using overall survival (OS).

### 2.5. Statistical Analysis

All analyses were conducted using SPSS Statistics v. 24.0 (IBM Corp., Armonk, NY, USA). Clinicopathological characteristics with categorical variables are presented as frequencies and proportions and were compared using the χ2 test. Continuous variables are expressed as means and standard deviations and were analyzed using the Student t test. To account for selection bias and unavoidable confounding factors, we performed a between-groups comparison by using IPTW with robust standard errors. A propensity score was estimated using multivariate logistic regression analysis involving nine covariates: age, sex, hypertension, diabetes mellitus, tumor location, T stage, N stage, metastasis pattern, and chemotherapy. Effect size estimates between the two groups after IPTW were presented by Cohen’s D. Long-term OS was estimated from the CRC diagnosis date to the last follow-up date using Kaplan–Meier methods and was compared using the log-rank test. The data of patients who survived were censored on December 31, 2018. Subgroup analysis of the risk factors for OS was conducted using IPTW-weighted Cox proportional hazards regression models. All statistical tests were two-tailed, and a *p* value of <0.05 was considered significant.

## 3. Results

### 3.1. Patient Characteristics

**Figure 2:**
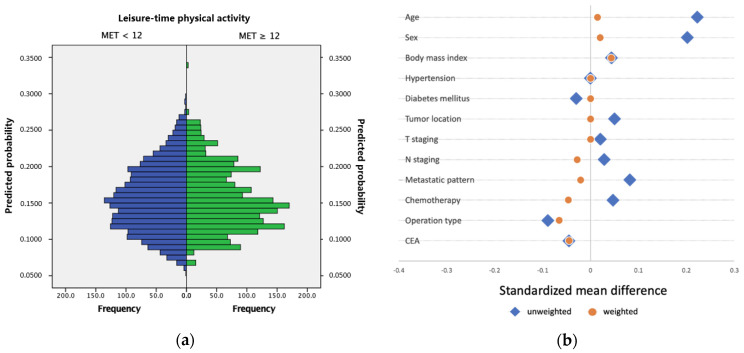
(**a**) Distribution of the propensity scores after inverse probability of treatment weighting (IPTW) for groups of weekly metabolic equivalent of task (MET) < 12 and MET ≥ 12. (**b**) Standardized mean differences in the unweighted and propensity score-weighted data analyses. Given the difference in baseline variables between the MET < 12 and MET ≥ 12 groups, a propensity score–based IPTW method was performed to balance the baselines of the two groups. After weighting, all between-group standardized mean differences were <0.1. Values of standardized mean differences between the two groups and the observed power for each test before and after weighting were shown in Table S1.

This study enrolled 2185 patients and divided them into two groups according to weekly MET values: MET < 12 group (*n* = 1845) and MET ≥ 12 group (*n* = 340). Before the IPTW method was applied, more patients in the MET < 12 group were younger (mean age: 60.11 ± 14.16 vs. 63.20 ± 12.37 years; *p* < 0.001) and female (45.7% vs. 35.6%; *p* < 0.001). After the propensity score–based IPTW was applied for age, sex, BMI, underlying diseases, tumor location, tumor stage, metastasis pattern, and chemotherapy administration, the propensity score distributions in both groups were balanced (Figure 2a). Imbalanced variables, including age and sex, were standardized (Figure 2b). No significant differences were found between the groups (Table 1).

### 3.2. Short-Term Outcomes

Table 2 revealed the short-term postoperative outcomes. After the IPTW process, the MET < 12 group showed a higher postoperative morbidity rate than the MET ≥ 12 group (18.7% vs. 10.6%; *p* < 0.001). The MET < 12 group had a higher rate of wound infection (3.5% vs. 1.5%; *p* < 0.001), lung complications (1.3% vs. 0.6%; *p* = 0.043), and gastrointestinal complications, such as postoperative ileus (4.2% vs. 1.8%; *p* < 0.001). There are no significant differences in cardiovascular-, urinary tract-, or intra-abdominal-related complications or anastomosis between the groups. The postoperative mortality rate was higher in the MET < 12 group than in the MET ≥ 12 group (2.4% vs. 0.6%; *p* < 0.001). In the severity of complications assessed by modified Clavien–Dindo classification, the MET < 12 group had a higher proportion of both low-grade (I/II) postoperative complications (12.8% vs. 7.4%; *p* < 0.001) and high-grade (III–V) complications (5.9% vs. 3.2%; *p* = 0.001) than the MET ≥ 12 group. The MET < 12 group experienced a longer postoperative hospital stay than the MET ≥ 12 group (12.75 ± 11.46 vs. 10.98 ± 6.12 days; *p* < 0.001).

### 3.3. Long-Term Outcomes and Prognostic Factor Analysis

**Figure 3:**
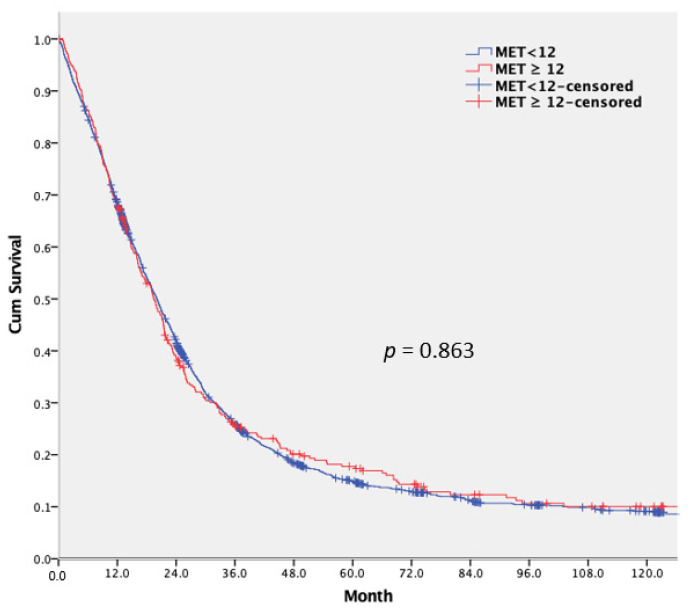
Relation between overall survival and weekly metabolic equivalent of task (MET; *p* = 0.863).

No significant differences were observed between the MET < 12 group and the MET ≥ 12 group. Figure 3 displays the Kaplan–Meier cumulative OS curves (*p* = 0.863). Weighted Cox proportional hazard regression analysis was performed to assess the associations between the multiple variables and the OS rate, and the results are displayed in Table 3. In the survival analysis for all patients, patients with the left-sided CRC (IPTW-adjusted hazard ratio [HR]: 0.772, 95% confidence interval [CI] = 0.691–0.861; *p* = 0.021), patients with a non-T4 tumor (IPTW-adjusted HR: 0.734, 95% CI = 0.586–0.919; *p* = 0.036), and patients who received R0 resection for the primary tumor and metastases (IPTW-adjusted HR: 0.455, 95% CI = 0.319–0.649; *p* = 0.023) had better overall survival.

## 4. Discussion

After completing the propensity score–based IPTW process for 2185 patients with stage IV CRC, we found that patients with MET ≥ 12 before operation had fewer postoperative complications/comorbidities and lower mortality rates than patients with MET < 12. No difference in OS was observed between the groups.

Among the complications observed, morbidities from wound infection, lung-related complications, gastrointestinal-related complications, and other rare complications were significantly higher in the MET < 12 group than in the MET ≥ 12 group. Furthermore, the severity of complications and the postoperative length of hospital stay were lower in the MET ≥ 12 group. Previous studies have revealed that physical activity is associated with a reduced risk of CRC. Studies have demonstrated that when comparing the most active and least active persons in regular physical activity, the CRC risk can be reduced by 25–30% [17,18]. However, studies on the association between preoperative physical activity and postoperative outcomes, especially short-term outcomes, are rare. Short-term outcomes include postoperative morbidities and their severity, postoperative mortality, and postoperative hospital stay duration. In our study, the overall complication rates (10.6% vs. 18.7%; *p* < 0.001) and mortality rates (2.4% vs. 0.6%; *p* < 0.001) were lower in the MET ≥ 12 group than in the MET < 12 group. One observational cohort study revealed that, for patients who underwent elective colorectal surgery, higher preoperative physical activity was related to faster self-assessed physical recovery; however, no significant difference observed in the length of hospital stay, readmittance, or reoperation [19]. One systemic review and meta-analysis revealed that a lower anaerobic threshold was associated with an increased risk of postoperative complications and was associated with a prolonged hospital stay [11].

Many different methods exist to evaluate patients’ preoperative physical activity. West et al. [20] used cardiopulmonary exercise testing for risk stratification and reported a significant difference in postoperative pulmonary-related and infection complications. Nutt et al. [21] used a preoperative shuttle walk test to determine the effect of physical activity on patients experiencing no complications versus patients developing complications and patients experiencing no/minor complications versus patients developing major complications. Lai et al. [22] reported that patients unable to perform a preoperative cardiopulmonary exercise test or those who demonstrate an anaerobic threshold had higher elective admissions, longer total hospital stay duration, and a higher mortality rate. Nikolopoulos et al. [23] demonstrated that cardiopulmonary exercise testing enabled the prediction of postoperative cardiopulmonary complications that resulted in a considerably longer median hospital stay duration; they found that spirometry was unable to be used for such a prediction. In our study, we used MET values to evaluate patients’ preoperative leisure-time physical activity levels. Patients in the MET ≥ 12 group had significantly fewer postoperative wound infections and lung and gastrointestinal complications than patients in the MET < 12 group. Postoperative hospital stay duration was also significantly shorter in the MET ≥ 12 group. This may be related to faster and more complete postoperative physical activity and ambulation recovery. Improved preoperative cardiopulmonary function likely also contributed to more favorable short-term outcomes.

Many studies have reported that physical activity is associated with more favorable long-term CRC outcomes [12,24,25,26]. However, these studies have evaluated stage I–III CRC patients. You et al. [12] reported that, for stage I-III CRC patients, preoperative leisure-time activity was significantly related to long-term outcomes including disease-free survival and OS. Phipps et al. [25] reported that in stage III colon cancer patients, better physical activity was linked to more favorable outcomes. Jayasekara et al. [26] reported that, for stage II CRC patients, physical activity was associated with ameliorated cancer-specific survival; nevertheless, no differences observed in stage I or stage III CRC patients. In our study, no significant difference was found in OS between the MET < 12 group and MET ≥ 12 group for patients with metastatic CRC (*p* = 0.863). Different viewpoints on the relation among metabolic processes, the immune system, and carcinogenesis exist to explain the link between physical activity and CRC outcomes. Brown et al. [27] demonstrated that aerobic exercise reduces visceral adipose tissue and may be a mechanism to reduce the risk of disease recurrence among CRC patients. Giovannucci et al. [28] suggested that physical activity and adiposity mainly operate through similar carcinogenic mechanisms. Pedersen [29] revealed a link among exercise, epinephrine, and interleukin-6 to natural killer cell mobilization, redistribution, and ultimately to the control of tumor growth. Slattery et al. [30] observed that high levels of physical activity reduced the risks of TP53 and KRAS2 rectal tumor mutations. However, in this study, physical activity had little impact on long-term survival in patients with metastatic CRC who underwent palliative resection.

Several risk factors affect the prognosis of patients with metastatic CRC. Carlomagno et al. [31] reported that peritoneal carcinomatosis and surgery of metastases independently affected survival in patients with metastatic CRC. Koerkamp et al. [32] demonstrated that the detection of circulating tumor cells in peripheral blood was associated with poor survival. Several mutational molecular markers including microsatellite instability, BRAF, KRAS/NRAS, and combination mutations were associated with worse outcomes. Riedl et al. [33] reported that inflammatory biomarkers such as the neutrophil-to-lymphocyte ratio; lymphocyte-to-monocyte ratio, and platelet-to-lymphocyte ratio were useful predictors of disease outcomes and treatment response in patients with metastatic CRC. Nozawa et al. [34] demonstrated that patients with metastatic CRC who underwent successful conversion to resection after chemotherapy had similar outcomes as patients with initially resectable stage IV CRC. Reed et al. [35] reported that diabetes or other metabolic syndrome elements were not prognostic factors for progression-free survival and OS in metastatic CRC. In our study, no difference was observed in long-term survival between the MET < 12 group and the MET ≥ 12 group. Therefore, physical activity should not be considered a prognostic factor for metastatic CRC.

This study has several limitations. First, this study had a retrospective design, which might have caused selection bias. Second, errors might have been present in the patient-reported MET values, which were relatively biased. A patient may identify slow walking or fast walking subjectively, and this can affect our MET calculation. In addition, it was restricted to conducting a dose-response study of MET values for all patients based on the current data. Third, the IPTW method was used to reduce imbalance and confounding effects between the groups; however, it also created a pseudopopulation, which might have caused some bias and residual confounding effects.

## 5. Conclusions

In conclusion, patients with metastatic CRC undergoing palliative resection with higher preoperative leisure-time physical activity exhibited more favorable short-term postoperative morbidity and mortality. However, no long-term survival difference was observed between the MET < 12 and MET ≥ 12 groups. Further investigation to evaluate the effect of postoperative physical activity on patients with metastatic colorectal cancer after palliative resection is warranted.

## Figures and Tables

**Table 1:** Patient demographic characteristics and clinicopathological features.

Variables	Overall *n* = 2185	MET < 12 *n* = 1845	MET ≥ 12 *n* = 340	*p* Value	IPTW-Adjusted *p* Value
Age, mean ± SD *	60.59 ± 13.941	60.11 ± 14.161	63.2 ± 12.374	<0.001	0.612
Sex, *n* (%) *				<0.001	0.483
Male	1220 (55.8)	1001 (54.3)	219 (64.4)		
Female	965 (44.2)	844 (45.7)	121 (35.6)		
BMI, kg/m^2^, *n* (%)				0.464	0.154
<25	1421 (69.9)	1205 (70.2)	216 (68.1)		
≥25	613 (30.1)	512 (29.8)	101 (31.9)		
missing	151				
Underlying illness, *n* (%) *					
Hypertension *				0.893	0.981
Yes	574 (26.3)	486 (26.3)	88 (25.9)		
No	1611 (73.7)	1359 (73.7)	252 (74.1)		
Diabetes mellitus *				0.484	0.823
Yes	287 (13.1)	247 (13.4)	40 (11.8)		
No	1898 (86.9)	1598 (86.6)	300 (88.2)		
Tumor location, *n* (%) *				0.723	0.876
Right-sided colon	603 (27.6)	504 (27.3)	99 (29.1)		
Left-sided colon	774 (35.4)	653 (35.4)	121 (35.6)		
Rectum	808 (37.0)	688 (37.3)	120 (35.3)		
T staging, *n* (%) *				0.762	0.804
non-T4	855 (39.1)	725 (39.3)	130 (38.2)		
T4	1330 (60.9)	1120 (60.7)	210 (61.8)		
N staging, *n* (%) *				0.456	0.226
N0	325 (14.9)	270 (14.6)	55 (16.2)		
N+	1860 (85.1)	1575 (85.4)	285 (83.8)		
Metastasis, *n* (%) *				0.227	0.555
Single organ	1329 (60.8)	1112 (60.3)	217 (63.8)		
Multiple organs	856 (39.2)	733 (39.7)	123 (36.2)		
Chemotherapy, *n* (%) *				0.582	0.382
Yes	1654 (75.7)	1392 (75.4)	262 (77.1)		
No	531 (24.3)	453 (24.6)	78 (22.9)		
Operation type, *n* (%)				0.867	0.217
Right colectomy	518 (23.7)	432 (23.4)	86 (25.3)		
Left hemicolectomy	90 (4.1)	74 (4.0)	16 (4.7)		
Anterior resection	1225 (56.1)	1034 (56.0)	191 (56.2)		
APR	79 (3.6)	68 (3.7)	11 (3.2)		
Segmental resection	59 (2.7)	50 (2.7)	9 (2.6)		
Subtotal or total	71 (3.2)	61 (3.3)	10 (2.9)		
Hartmann operation	143 (6.5)	126 (6.8)	17 (5.0)		
CEA (ng/mL), *n* (%)				0.459	0.191
<5	574 (26.9)	479 (26.6)	95 (28.6)		
≥5	1560 (73.1)	1323 (73.4)	237 (71.4)		
missing					

MET: metabolic equivalent of task; IPTW: inverse probability of treatment weighting; BMI: body mass index; APR: abdominoperineal resection; SD: standard deviation; CEA: Carcinoembryonic Antigen; * Variables used for the propensity score weighting process.

**Table 2:** Comparison of short-term outcomes between the weekly MET < 12 and MET ≥ 12 groups after weighting.

Variables	MET < 12 *n* = 1845	MET ≥ 12 *n* = 340	IPTW-Adjusted *p* Value	OR (95% CI)
Postoperative morbidities, *n* (%)				
Yes	345 (18.7)	36 (10.6)	<0.001	0.532 (0.448–0.632)
No	1500 (81.3)	304 (89.4)		
Type of complications, *n* (%)				
Wound infection	65 (3.5)	5 (1.5)	<0.001	0.377 (0.247–0.578)
Lung	24 (1.3)	2 (0.6)	0.043	0.498 (0.262–0.949)
Cardiovascular	8 (0.4)	1 (0.3)	0.607	0.668 (0.237–1.88)
Urinary tract	57 (3.1)	10 (2.9)	1.0	1.004 (0.713–1.412)
Gastrointestinal	77 (4.2)	6 (1.8)	<0.001	0.383 (0.259–0.565)
Intraabdominal	21 (1.1)	2 (0.6)	0.072	0.519 (0.265–1.017)
Anastomosis	34 (1.8)	3 (0.9)	0.062	0.607 (0.368–1.003)
Others	37 (2.0)	4 (1.2)	0.026	0.555 (0.336–0.918)
Postoperative mortality, *n* (%)				
Yes	44 (2.4)	2 (0.6)	<0.001	0.304 (0.173–0.533)
No	1801 (97.6)	338 (99.4)		
Modified Clavien–Dindo classification				
Low grade (I/II)	236 (12.8)	25 (7.4)	<0.001	0.53 (0.432–0.65)
High grade (III/IV/V)	109 (5.9)	11 (3.2)	0.001	0.619 (0.466–0.821)
Postoperative hospital stay, (day)				
Mean (±SD)	12.75 (11.463)	10.98 (6.116)	<0.001	
Median	10.0	9.0		

MET: metabolic equivalent of task.

**Table 3:** Multivariable models from weighted Cox proportional hazard regression for overall survival.

Variables	IPTW-Adjusted		
	Hazard Ratio	95% CI	*p* Value
Age			
<65	0.847	0.611–1.175	0.098
≥65	1		
Gender			
female	1.028	0.847–1.248	0.319
male	1		
BMI, kg/m^2^			
<25	1.024	0.605–1.735	0.668
≥25	1		
Hypertension			
No	1.022	0.056–18.806	0.939
Yes	1		
Diabetes mellitus			
No	1.029	0.239–4.437	0.846
Yes	1		
Tumor location			
Right-sided colon	0.971	0.617–1.528	0.563
Left-sided colon	0.772	0.691–0.861	0.021
Rectum	1		
T staging			
non-T4	0.734	0.586–0.919	0.036
T4	1		
N staging			
N0	0.62	0.305–1.26	0.074
N+	1		
Metastasis			
Single organ	0.718	0.09–5.747	0.293
Multiple organs	1		
Chemotherapy			
No	2.579	0.753–8.837	0.065
Yes	1		
Primary and metastatic tumor resection			
R0 resection	0.455	0.319–0.649	0.023
Non-R0 resection	1		
CEA (ng/mL)			
<5	0.802	0.446–1.441	0.131
≥5	1		

MET: metabolic equivalent of task; IPTW: inverse probability of treatment weighting; BMI: body mass index; CI: confidence interval; CEA: Carcinoembryonic Antigen.

## Data Availability

Data available on request due to restrictions. The data presented in this study are available on request from the corresponding author. The data are not publicly available due to privacy.

## Supplementary Materials

The following are available online at https://www.mdpi.com/article/10.3390/cancers14030489/s1, Table S1: Values of standardized mean differences in the unweighted and propensity score-weighted data analyses between MET < 12 and MET ≥ 12.
