# Supplementary material for: Association of Preoperative Physical Activity with Short- and Long-Term Outcomes in Patients Undergoing Palliative Resection for Metastatic Colorectal Cancer: An Inverse Probability of Treatment Weighting Analysis
Source: Cancers (Basel). 2022 Jan 19;14(3):489. doi: 10.3390/cancers14030489 (PMC8833797; doi:10.3390/cancers14030489)
Supplement: Supplementary file 1 [file cancers-14-00489-s001.zip › cancers-1519058-supplementary.pdf]

cancers

MDPI

Association of Preoperative Physical Activity with Short- and Long-Term Outcomes in Patients Undergoing Palliative Resection for Metastatic Colorectal Cancer: An Inverse Probability of Treatment Weighting Analysis

Ching-Chung Cheng, I-Li Lai, Shu-Huan Huang, Wen-Sy Tsai, Pao-Shiu Hsieh, Chien-Yuh Yeh, Sum-Fu Chiang, Hsin-Yuan Hung and Jeng-Fu You

Table S1. Values of standardized mean differences in the unweighted and propensity score-weighted data analyses between MET < 12 and MET ≥ 12.

Variables	Before IPTW-Weighting		After IPTW-Weighting	
	SMD †	Observed Power ‡	SMD †	Observed Power ‡
Age	0.2223	0.789	0.0147	0.078
Sex	0.202	0.935	0.0202	0.351
BMI, kg/m^2	0.0435	0.112	0.0433	0.265
Hypertension	0	0.054	0	0.054
Diabetes mellitus	−0.0296	0.129	0	0.051
Tumor location	0.0501	0.102	0	0.084
T staging	0.0205	0.066	0	0.071
N staging	0.0281	0.114	−0.0276	0.267
Metastatic pattern	0.082	0.234	−0.0205	0.156
Chemotherapy	0.0466	0.227	−0.0462	0.334
Operation type	−0.0892	0.076	−0.0651	0.264
CEA (ng/mL)	−0.0451	0.120	−0.0449	0.225

MET = metabolic equivalent of task; IPTW = inverse probability of treatment weighting; BMI = body mass index; SMD = standardized mean difference. † Cohen presented that d = 0.2 is considered a 'small' effect size, 0.5 represents a 'medium' effect size, and 0.8 express a 'large' effect size, which means that if the difference of means between two groups is less than 0.2 standard deviations, the difference is insignificant. ‡ Computed using alpha = 0.05.
